# Oxidative Stress and Microcirculatory Flow Abnormalities in the Ventricles during Atrial Fibrillation

**DOI:** 10.3389/fphys.2012.00236

**Published:** 2012-07-05

**Authors:** Andreas Goette, Alicja Bukowska, Christopher H. Lillig, Uwe Lendeckel

**Affiliations:** ^1^Department of Cardiology and Intensive Care Medicine, St. Vincenz-Hospital PaderbornPaderborn, Germany; ^2^Working Group: Molecular Electrophysiology, University Hospital MagdeburgMagdeburg, Germany; ^3^Institute of Medical Biochemistry and Molecular Biology, Ernst-Moritz-Arndt UniversityGreifswald, Germany

**Keywords:** angiotensin, atrial fibrillation, microvascular flow, oxidative stress

## Abstract

Patients with atrial fibrillation (AF) often present with typical angina pectoris and mildly elevated levels of cardiac troponin (non-ST-segment elevation myocardial infarction) during an acute episode of AF. However, in a large proportion of these patients, significant coronary artery disease is excluded by coronary angiography, which suggests that AF itself influences myocardial blood flow. The present review summarizes the effect of AF on the occurrence of ventricular oxidative stress, redox-sensitive signaling pathways and gene expression, and microcirculatory flow abnormalities in the left ventricle.

## Introduction

Angina pectoris is a typical symptom in patients with paroxysmal atrial fibrillation (AF). In most of these patients, angina pectoris is associated with mildly elevated cardiac troponin (cTn) levels suggesting a non-ST-segment elevation myocardial infarction (NSTEMI). However, in a large proportion of these patients, significant coronary artery disease can be excluded by coronary angiography despite clinical symptoms (Fuster et al., 2006; Brown et al., 2007). Although the elevated ventricular rate during AF may contribute to the symptoms of angina pectoris (Fuster et al., 2006), angina pectoris develops also in patients with a slow ventricular rate and most patients tolerate fast ventricular rates in sinus rhythm without any clinical symptoms (Van Gelder et al., 2002; Wyse et al., 2002). Recent reports suggest that myocardial blood flow is reduced, whereas coronary vascular resistance is elevated in patients with AF (Kochiadakis et al., 2002; Range et al., 2007). One potential link between AF, abnormal ventricular perfusion, and cardiomyocyte dysfunction is the occurrence of oxidative stress and the disruption of redox signaling through activation of the nicotinamide adenine dinucleotide phosphate oxidase (NADPH oxidase; Kern et al., 2006; Camici and Crea, 2007; Doughan et al., 2008). Repetitive episodes of AF-induced ventricular ischemia may contribute to the development of a pathological vicious cycle combining AF and left ventricular (LV) dysfunction.

## Ventricular Oxidative Stress and Signal Transduction during AF

**Figure 1:**
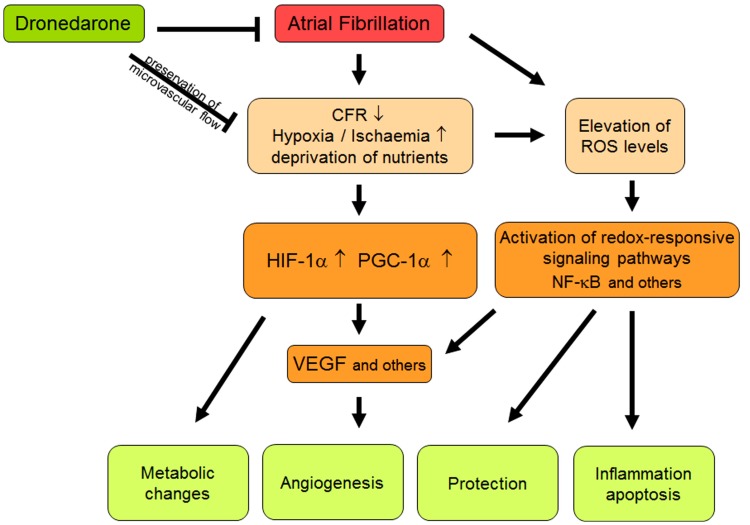
**Schematic summary of different interactions between atrial fibrillation, oxidative stress, and flow abnormalities**. Abbreviations should be included here are explained in the text.

Reactive oxygen species (ROS) are generated under physiological conditions in the cardiovascular system and act as second messengers in numerous redox-sensitive signal transduction pathways (Figure 1). However, under pathophysiological conditions, chronically elevated amounts of ROS may exert oxidative stress. Historically, the term “oxidative stress” was defined as an imbalance between the generation of ROS and the capacity of the defense systems (Cadenas et al., 1982). During the past decade, this model has evolved based on some key findings: the production of different oxidants affects distinct presets of target proteins through modifications that are specific both with respect to the oxidant and the site of modification, most frequently well-defined cysteinyl side chains. The so-called antioxidant redox systems in the different cellular compartments, e.g., glutathione, NADPH, thioredoxin (Trx), and peroxidases such as the peroxiredoxins (Prx), are, however, not in equilibrium and independently maintained at distinct redox potentials. Oxidative stress may thus, more timely, be defined as the chronic disturbance of redox circuits and redox-responsive signal transduction pathways (Ghezzi et al., 2005;Jones, 2006, 2008).

Oxidative stress has been implicated as playing a critical role in the pathophysiology of heart and cardiovascular diseases such as heart failure, LV hypertrophy, coronary heart disease, cardiac arrhythmia. It appears that the oxidative events initiate the disease-dependent tissue remodeling and promote its propagation. Oxidative stress is associated with microvascular flow abnormalities and occurs immediately after new-onset AF likely representing key initiator mechanisms of AF-related ventricular remodeling. This has been shown in patients with lone recurrent AF and for rapid atrial pacing models (Kochiadakis et al., 2002; Bukowska et al., 2008, 2012; Goette et al., 2009). Irregular RR intervals are considered to be responsible for compromised coronary blood flow reserve, thus preventing the adequate attainment of the increased oxygen demand due to tachycardia (Kochiadakis et al., 2002). In addition, increased catecholamine levels via α-adrenergic vasoconstriction may further contribute to AF-induced ischemia (Heusch et al., 2000; Heusch, 2008). AF-dependent cardiac remodeling, especially fibrosis, may well contribute to long-term restriction of microcirculation (De Boer et al., 2003). Resulting imbalances of myocardial oxygen supply and myocardial oxygen demand lead to the specific activation of redox-sensitive signaling pathways, that are either protective or part of the pathophysiological process causing onset and progression of AF.

Ischemia facilitates the onset of AF by altering cellular ionic homeostasis, in particular via tachycardia-induced intracellular calcium and sodium overload. Increased spontaneous ectopy is due to increased NCX currents and spontaneous Ca^2+^-release events (Nishida et al., 2011). In pulmonary veins (PV), hypoxia-induced EAD, and delayed after-depolarizations (DAD) as well as reoxygenation-induced PV burst firing represent important proarrhythmogenic mechanisms (Lin et al., 2012).

Coronary flow can be estimated *in vivo* using wire-based systems (Goette et al., 2009; Bukowska et al., 2012). Coronary flow reserve (CFR) measurements can be measured using a pressure temperature sensor-tipped guidewires, which allow the simultaneous determination of the fractional flow reserve (FFR). CFR measurements are influenced by flow abnormalities in the epicardial arteries and the microcirculation. In contrast, reduced FFR is specific to epicardial lesions. Recent studies can clearly show that acute episodes of AF induce oxidative stress in the LV myocardium and compromise microvascular blood flow (Goette et al., 2009; Bukowska et al., 2012).

Although it is well established that ischemia creates a substrate for AF maintenance (Sinno et al., 2003; Rivard et al., 2007), the effects of AF on ventricular microcirculation and underlying pathways are less well understood. Impaired intracellular Ca^2+^-handling as described above together with elevated cardiac and systemic angiotensin II levels are two important factors which very likely contribute to the AF-dependent activation of redox-sensitive signaling pathways in the ventricles. These will be discussed in more detail below.

### Vasoconstrictory peptides regulating coronary flow

#### Angiotensin II

Vasoactive peptides such as angiotensin II (AngII) and endothelin (ET) play important roles in the regulation of cardiovascular function as well as in the pathogenesis of heart and cardiovascular diseases. AF has been associated with increased plasma and tissue levels of angiotensin II (AngII) (Cardin et al., 2003; Goette et al., 2008), which are resulting from increased expression/activity of ACE (Goette et al., 2000).

Angiotensin II mediates its major hemodynamic and pathophysiological effects via the AT1 receptor. Six hours of rapid atrial pacing is sufficient to elevate plasma AngII levels suggesting the very early involvement of this vasoactive molecule in the pathogenesis of AF (Goette et al., 2008). The activation of the AT1 receptor induces a cascade of phosphorylation events that eventually activates MAP kinases, which stimulate the proliferation of fibroblasts, cellular hypertrophy, and apoptosis. Furthermore, activation via the AT1 receptor releases calcium from intracellular stores and activates protein kinase C (PKC). PKC regulates the function of calcium and potassium channels, and phosphorylates p47^phox^ regulatory subunit. The phosphorylation of p47^phox^ plays a pivotal role in the activation of NOX2/NOX1 by providing physical binding domains to another regulatory subunit p67^phox^ (Fontayne et al., 2002). It is noteworthy that both the MAP kinase as well as the calcium/PKC signaling pathways respond to ROS themselves and are regulated by redox effector molecules from the Trx family of proteins (Berndt et al., 2007; Matsuzawa and Ichijo, 2008).

At the atrial level, it is well established that AngII upon binding to its preferred receptor, AT1R, leads to the activation of NADPH oxidase (Oudot et al., 2003; Doughan et al., 2008; Goette et al., 2009). In blood vessels, AngII infusion resulted in increased expression and activity of NADPH oxidase, which was dependent on PKC to some extent (Mollnau et al., 2002). In the murine heart, AngII increased superoxide generation and cardiac hypertrophy involving NOX2 (Bendall et al., 2002). Increased NADPH oxidase activity contributes to elevated ROS production, protein modification, and redox-related gene expression patterns observed in AF (Carnes et al., 2001). Moreover, it has been identified as an independent risk factor for post-operative AF (Kim et al., 2008). Pre-operative treatment with ascorbate prevented AF to a significant extent (Carnes et al., 2001). Similarly, ascorbate reduced the recurrence of AF after cardioversion (Korantzopoulos et al., 2005).

Less is known about the effects of AF on ventricular function and underlying changes in redox-signaling pathway activity and related gene expression. AF is associated with increased coronary resistance, compromised vasodilation (Takahashi et al., 2002), reduced coronary flow (Range et al., 2007), or flow reserve (Goette et al., 2009; Bukowska et al., 2012). As these changes can be largely attenuated by the administration of sartans (Goette et al., 2009). AngII seems to be a major factor linking AF with LV malperfusion and dysfunction. In support of this view, the RAP-induced Ang-II-dependent activation of NADPH oxidase and down-stream effectors of redox-activated signaling cascades, e.g., LOX-1 expression and F_2_-isoprostane formation, were all attenuated by irbesartan (Goette et al., 2009).

At the molecular level, ANG-II-receptor blockers (ARBs) have been shown to increase NO availability and it is possible that this effect is mediated by a stronger ANG-II-dependent activation of AT_2_-receptors. ARBs also attenuate aortic intimal proliferation and markedly decrease the enhanced LOX-1 expression in the aorta of hypercholesterolemic animals (Chen et al., 2000). In a recent study it was shown that application of irbesartan prevents ventricular oxidative stress and microvascular flow abnormalities during 7 h of AF (Goette et al., 2009). Nevertheless, clinical trials like the ACTIVE study failed to demonstrate a measurable benefit of long-term ARB therapy in patients with AF (ACTIVE I Investigators et al., 2011).

#### Endothelin-1

Endothelin-1 (ET-1) exerts its proarrhythmogenic effects by two different ways. First, due to its strong vasoconstrictory activity, ET-1 may induce ischemia which facilitates arrhythmia. Second, upon binding to endothelin receptor A (ET-A), ET-1 affects intracellular calcium handling and, in particular, provokes intracellular Ca^2+^-waves via IP3-dependent Ca^2+^-release leading to DAD (Li et al., 2005). Furthermore, ET-1 activates PKC and MAP kinases (Sugden, 2003). Atrial stretch is a potent factor promoting the production and release of ET-1 (Bruneau et al., 1997). ET-1 activates NADPH oxidase via the ET receptor-proline-rich tyrosine kinase-2 (Pyk2)-*rac1* pathway (Dammanahalli and Sun, 2008). The GTPase, *rac1*, binds to p67^phox^ and activates NADPH oxidase in its GTP-bound state (Rinckel et al., 1999).

Heart failure (Galatius-Jensen et al., 1996; Zolk et al., 1999; Love et al., 2000; Mayyas et al., 2010), valvular disease (Kinoshita et al., 1993), primary pulmonal hypertension (Rubens et al., 2001), but also AF are all associated with increased plasma and cardiac tissue levels of ET-1. Atrial ET-1 levels were correlated with atrial rhythm, atrial size, and hypertension and were associated with hypertrophy, fibrosis, and atrial dilatation (Mayyas et al., 2010). Changes in cardiac and circulating levels of ET-1 have been described in experimental models of myocardial ischemia and in patients with acute myocardial infarction (Hasdai et al., 1994; Brunner et al., 1997; White et al., 2001). Accordingly, a dual ET receptor antagonist has been demonstrated to prevent coronary vasoconstriction during reperfusion of ischemic heart (Besse et al., 2001). AF-dependent negative changes in the microcirculation (Goette et al., 2009; Bukowska et al., 2012) contribute to and further enhance increased gene expression and release of ET-1 which, in turn, aggravates coronary vasoconstriction (Neubauer et al., 1991; Hiller et al., 1997) and leads to oxidative stress (Nagase et al., 1990). Although the AngII/ET-1-induced increase of ROS generation is mostly associated with hemodynamic response and development of hypertension, solid evidence shows that these vasoactive peptides via activation of NADPH oxidase mediate changes in vascular architecture and heart damage (Amiri et al., 2004; Zhang et al., 2005).

#### NADPH oxidase

It has been suggested that the NADPH oxidase is an important source of ROS in the left ventricle during atrial tachyarrhythmia (Goette et al., 2009; Bukowska et al., 2012). NADPH oxidase was originally discovered in neutrophils, where, during phagocytosis, millimolar quantities of superoxide can be released into the extracellular (phagosomal) compartment. In non-phagocytic cells such as cardiomyocytes, fibroblasts, and endothelial cells, the amounts of produced superoxide are much lower and occur mostly intracellularly (Li and Shah, 2003). The neutrophil NADPH oxidase is composed of membrane-associated subunits: p22^phox^, and NOX, and four cytosolic regulatory subunits: p47^phox^, p67^phox^, p40^phox^, and the small GTPase *rac1* or *rac2*. NOX is the key catalytic subunit of the NADPH oxidase and in non-phagocytic cells possess several isoforms. Beside the phagocyte NADPH oxidase (NOX2; gp91^phox^), the expression of six homologs (NOX1, NOX3, NOX4, NOX5, Duox1, and Duox2) has been identified (Afanas’ev, 2011). While the cytosolic regulatory components translocate to the membrane to form the active NADPH oxidase complex upon activation in the neutrophil (Babior et al., 2002), in the non-phagocytic cells preassembled functional enzyme complex is partly present intracellularly (Bayraktutan et al., 2000; Li and Shah, 2003). Although the non-phagocyte NADPH oxidase is constitutively active, its activity can be further up-regulated in response to vasoactive peptides (AngII, ET-1), hormones, growth factors, cytokines, and mechanical stress (Jaimes et al., 1998; Griendling et al., 2000; Li et al., 2002; Yasunari et al., 2002).

The NOX-dependent ROS signaling is an important factor responsible for development of many pathological processes in the ventricles during cardiac hypertrophy, remodeling, and heart failure (Murdoch et al., 2006). Recently, in an animal model of acute AF, increased expression of NOX2, NOX1, and enhanced expression of NOX4 were shown in the left ventricle after 6 h of atrial pacing (Goette et al., 2009; Bukowska et al., 2012). The AT1 receptor antagonist, irbesartan, and the multichannel inhibitor, dronedarone, efficiently prevented the up-regulation of NOX2 (Goette et al., 2009; Bukowska et al., 2012). The elevated expression of NOX2 and superoxide production in the left ventricle was also observed in a rabbit model of chronic heart failure (Yasunari et al., 2002). Several studies have shown a crucial role of NOX2 in the response to AngII-induced LV hypertrophy (Bendall et al., 2002; Li and Shah, 2003; Li et al., 2007). It was found that the regulatory compound *rac1* initiated hypertrophic response (Hingtgen et al., 2006; Satoh et al., 2006). Amounts of the regulatory p47^phox^ (Hingtgen et al., 2006; Satoh et al., 2006) increase after myocardial infarction in the left ventricle and contribute to the NADPH oxidase dependent tissue remodeling (Doerries et al., 2007). NOX2 and NOX4 are the main isoforms expressed in the cardiac cells. NOX4, in contrast to NOX2, does not require the presence of regulatory oxidase proteins p47^phox^ or the GTPase *rac*. Moreover, NOX4 produces mainly hydrogen peroxide and only very small amounts of superoxide intracellularly (Serrander et al., 2007) and was found on internal membranes, in mitochondria (Ago et al., 2010; Kuroda et al., 2010), and also in perinuclear endoplasmic reticulum (Chen et al., 2008). The up-regulation in NOX4 expression was accompanied by mitochondrial dysfunction and apoptosis in the cardiomyocytes (Ago et al., 2010; Kuroda et al., 2010). NOX1 is an important isoform expressed particularly in vascular smooth muscle cells and is responsible for extracellular superoxide production in coronary arterial myocytes. Several studies have provided evidence that NOX1 oxidase is involved in mediating the hypertensive response to AngII in particular (Dikalova et al., 2005; Matsuno et al., 2005).

Recent data suggests, however, that induction of atrial NADPH oxidase activity or subunit expression is an early but transient mechanism in the natural course of AF development and progression (Reilly et al., 2011). With increasing duration of AF, ROS production is shifted from NADPH oxidase to mitochondrial oxidases and uncoupled eNOS in the right atrium (Reilly et al., 2011). This is in full accordance with the observation that statins, which reduce ROS production by NADPH oxidases via inhibition of Rac1, are effective in acute models of AF and in patients with post-operative AF, but fail to reduce ROS production in models of long-lasting AF or patients with permanent AF. Pre-operative statin-treatment was shown to reduce myocardial O_2_^−^ and ONOO^−^ production by reducing NADPH oxidase activity (Antoniades et al., 2012).

## Ventricular Microcirculation during AF

**Figure 2:**
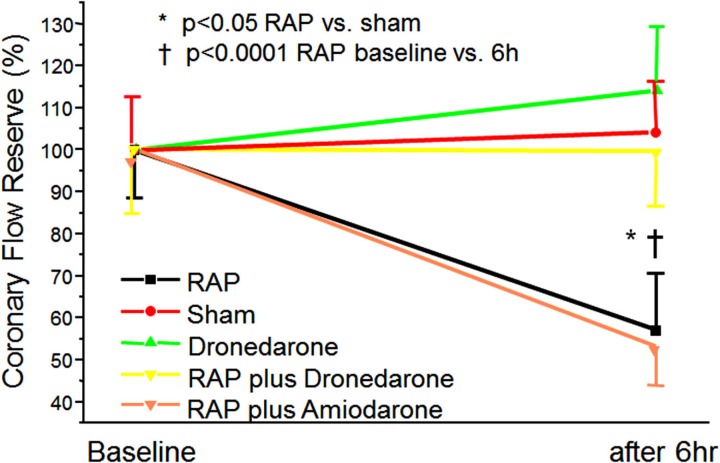
**Induction of ventricular-flow abnormalities in the ventricles during rapid atrial pacing (RAP) in comparison to unpaced controls using a porcine model**. Effects of dronedarone and amiodarone demonstrated. Abbreviations are explained in the text adopted from Bukowska et al. (2012).

An induced episode of AF of up to 6 h has no effect on FFR (marker for epicardial flow) in pigs (Bukowska et al., 2012). In contrast, CFR (index of microvascular abnormalities if FFR is normal) is substantially reduced (about 50%) after an AF episode of 6 h. Interestingly, application of irbesartan and dronedarone could prevent microcirculatory flow abnormalities to occur whereas amiodarone has no effect on CRF (Figure 2). These results correspond to latest findings that dronedarone reduces the size and volume of induced cerebral and myocardial infarcts (Engelhorn et al., 2011; Skyschally and Heusch, 2011). Patients without previously documented coronary artery disease sometimes develop chest discomfort with the onset of AF (Fineschi et al., 2008). Furthermore, patients with AF have ventricular-flow abnormalities and a higher incidence of cardiac events (Abidov et al., 2004; Range et al., 2007). Consistent with this notion, coronary artery resistance is markedly elevated (by 62%), whereas myocardial blood flow is substantially reduced in AF patients (Range et al., 2007). Vasodilatation in response to exercise is also compromised during AF (Berndt et al., 2007). The Doppler-derived coronary vascular resistance index has been reported to be increased by 67% in an experimental AF model (Range et al., 2007). Induction of AF for up to 6 h has no effect on FFR (marker of epicardial flow) in pigs (Bukowska et al., 2012); by contrast, CFR (index of microvascular abnormalities if FFR is normal) is substantially reduced (about 50%) by short-term AF. Interestingly, irbesartan and dronedarone could prevent the occurrence of microcirculatory flow abnormalities whereas amiodarone had no effect (Figure 2). These results are in keeping with recent findings indicating that dronedarone reduces the size and volume of induced cerebral and myocardial infarcts (Engelhorn et al., 2011; Skyschally and Heusch, 2011).

## AF Alters Ventricular Expression of Ischemia/Hypoxia-Related Gene Panels

Atrial fibrillation provokes rapid and profound changes in the ventricular expression of ischemia/hypoxia-related genes (Bukowska et al., 2012). These expression changes were associated with and may result partially from microcirculatory abnormalities. Both the observed RAP-dependent limitation of flow reserve and the expression changes could be prevented by dronedarone. Interestingly, amiodarone does not reduce AF-induced flow abnormalities in the microvascular tree (Figure 2). The positive effect of dronedarone in brief episodes of AF is supported by the ATHENA trial, which found a reduced rate of acute coronary syndromes and reduced cardiovascular mortality in patients with AF (Hohnloser et al., 2009).

**Figure 3:**
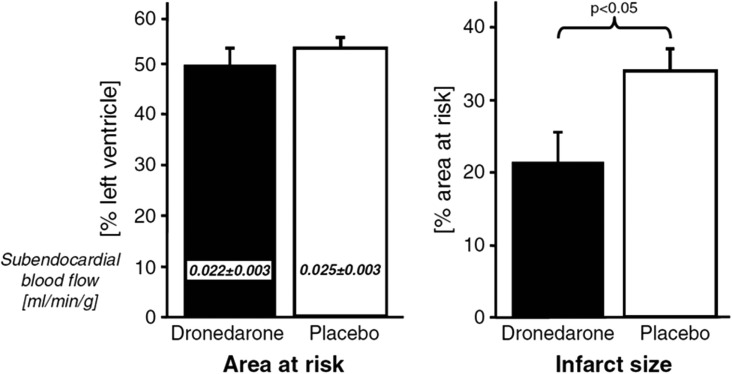
**Effect of dronedarone on size of acute myocardial infarctions adopted from Qiu et al. (2006)**.

A recent study could show that acute application of dronedarone during an induced myocardial infarction reduced the infarct size substantially (Figure 3). Nevertheless, in long-lasting (6 month) AF, myocardial NADPH is not activated (Reilly et al., 2011), and thereby, positive effects through inhibition of NADPH are unrealistic to exist. Thus, the antioxidant effects of dronedarone should not be present in permanent AF. This is supported by the PALLAS trial, which showed negative outcome if patients with permanent AF are treated with dronedarone. In PALLAS, rates of stroke, myocardial infarction, and heart failure were almost doubled in dronedarone treated patients (Connolly et al., 2011). Thus, the therapeutic effect of dronedarone depends on the duration of AF, which is quite a unique finding.

Transcriptome analyses provided a first mechanistic insight into the pathophysiological processes mediating or even counter-acting coronary and ventricular dysfunction during AF. Among the genes the expression of which was changed in response to RAP were HIF-1, VEGFA, and PPARGC1α, all of them known to be induced in response to hypoxia or deprivation of nutrients (Arany et al., 2008). The regulation of  VEGF expression in response to hypoxia is mediated by HIF-1α (Ferrara et al., 2003). Under the same conditions, and independent of this canonical HIF-pathway, increased PPARGC1α exerts its strong angiogenic activity and induces VEGF expression by co-activating ERR-α (Arany et al., 2008). Thus, both HIF-1α and PPARGC1α appear to be critically involved in the angiogenic response to AF-dependent flow alterations and may provide protection against ischemic damage.

Rapid atrial pacing also led to an activation of the NF-κB pathway in the left ventricle (Bukowska et al., 2012). ROS as intracellular messengers and redox effector molecules such as Trx and glutaredoxin (Grx) lead to the activation and nuclear translocation of this redox-sensitive transcription factor (Lillig and Holmgren, 2007). Consistent with the RAP-dependent ventricular activation of NF-κB, the expression of a panel of established down-stream targets of NF-κB including VEGFA (Kiriakidis et al., 2003; Martin et al., 2009), Fn14, CCL2 (Lawrence et al., 2006), HIF1A (Kunsch and Medford, 1999; Bonello et al., 2007) as well as DnaJ family members, DNAJA4 and DNAJB9, that have been described as co-chaperones for the ATPase activity of Hsp70 and function to protect stressed cells from apoptosis (Qiu et al., 2006), was up-regulated in the left ventricle (Bukowska et al., 2012). Both DNAJA4 and DNAJB9, but also thioredoxin (Trx1; Bloom and Jaiswal, 2003) and peroxiredoxin I (PrxI; Ago et al., 2008) are antioxidant response element (AREs) regulated genes activated through nuclear factor-erythroid 2-related factor 2 (Nrf2) in response to oxidative stress. After phosphorylation by, e.g., PKC, Nrf2 translocates to the nucleus where it binds to AREs and transactivates target genes of, e.g., enzymes such as PrxI that regulate the intracellular amounts of ROS (Bloom and Jaiswal, 2003). It seems reasonable to assume that increased expression of anti-oxidative response genes, e.g., peroxiredoxins and DnaJ family members, is aimed at limiting stress-mediated tissue-damage. In this *in vivo* model of acute AF, dronedarone attenuated most of the ventricular changes in gene expression. In addition, RAP-dependent PKC phosphorylation, NADPH isoform expression, isoprostane release, and IκBα phosphorylation were decreased. This, together with the attenuation of negative flow alterations may indicate that dronedarone beneficially affects very early steps of RAP-associated ventricular pathology, very likely by preventing ischemia/hypoxia itself.

PPARGC1, the multi-functional co-activator, is also involved in the regulation of cardiac mitochondrial functional capacity and cellular energy metabolism. In accordance with the observed increase in PPARGC1 and HIF-1α expression, RAP provoked profound changes in the ventricular expression of important metabolic genes including hexokinase 2 (HK2), glycogen synthase kinase 3β (GSK-3β), muscle isoform of glycogen phosphorylase (PYGM), and acyl-coenzyme A dehydrogenase (ACADL; Bukowska et al., 2012). Interestingly, these metabolic changes were not affected by dronedarone, which suggests that other factors than deprivation of oxygen and nutrients contribute to the overall change of ventricular gene expression during AF.

It is fully established that even mild ischemia is associated with compromised mitochondrial function and requires metabolic adaption to maintain adequate ATP generation and cardiac output (Shohet and Garcia, 2007). Again, the activation of redox-sensitive transcription factors, namely HIF-1α and PPARGC1, is responsible for these protective changes in the metabolism that generally mediate the shift from aerobic metabolism and fatty acid utilization to glucose utilization via pyruvate oxidation or even to glycolytic metabolism (Bolukoglu et al., 1996; Seagroves et al., 2001). HIF-1, together with c-myc, mediates the induction of HK2 which contributes to shift glucose away from mitochondrial utilization and has also anti-oxidative effects (Ahmad et al., 2002; Kim et al., 2006). On this background, the observed ventricular induction of glycolytic gene expression during AF completely fits to the increased expression levels of HIF-1α and PPARGC1 and, most importantly, demonstrate that the compromised microcirculatory flow leads to ischemia-like conditions. The latter activate multiple signaling pathways that are aimed at the improvement of oxygen supply, angiogenesis, cell survival, and adaption of metabolism (Figure 1).

## Conclusion

AT induces oxidative stress in the atrial and ventricular myocardium. In the ventricles, AF causes alterations in gene expression and activation of specific signal transduction pathways. As a consequence, microcirculation is impaired, troponin can be released, which is associated with causing clinical symptoms like angina pectoris and dyspnea. It remains to be determined if these alterations are also related to the increased rate of death in AF patients shown by several epidemiologic studies.

## Conflict of Interest Statement

The authors declare that the research was conducted in the absence of any commercial or financial relationships that could be construed as a potential conflict of interest.
